# Biotransformation of oral contraceptive ethynodiol diacetate with microbial and plant cell cultures

**DOI:** 10.1186/1752-153X-6-109

**Published:** 2012-09-29

**Authors:** Salman Zafar, Sammer Yousuf, Hammad A Kayani, Saifullah, Saifullah Khan, Abdullah M Al-Majid, M Iqbal Choudhary

**Affiliations:** 1 H. E. J. Research Institute of Chemistry, International Center for Chemical and Biological Sciences, University of Karachi, Karachi, 75270, Pakistan; 2 Department of Chemistry, Abdul Wali Khan University, Mardan, 23200, Pakistan; 3 Department of Chemistry, College of Science, King Saud University, PO Box 2455, Riyadh, 11451, Saudi Arabia

**Keywords:** Ethynodiol diacetate, Microbial transformation, Biotransformation, *Ocimum basilicum*, *Azadirachta indica*, *Cunninghamella elegans*, Cell suspension culture, Norethisterone, 17α-Ethynylestr-4-en-3β,17β-diacetoxy-6α-ol, 17α-Ethynylestr-4-en-3β,17β-diacetoxy-6β-ol, 17α-Ethynylestr-4-en-3β,17β-diacetoxy-10β-ol

## Abstract

**Background:**

Biotransformation by using microbial and plant cell cultures has been applied effectively for the production of fine chemicals on large scale. Inspired by the wealth of literature available on the biotransformation of steroids, we decided to investigate the biotransformation of ethynodiol diacetate (**1**) by using plant and microbial cultures.

**Results:**

The biotransformation of ethynodiol diacetate (**1**) with *Cunninghamella elegans* and plant cell suspension cultures of *Ocimum basilicum* and *Azadirachta indica* is being reported here for the first time. Biotransformation of **1** with *Cunninghamella elegans* yielded three new hydroxylated compounds, characterized as 17α-ethynylestr-4-en-3β,17β-diacetoxy-6α-ol (**2**), 17α-ethynylestr-4-en-3β,17β-diacetoxy-6β-ol (**3**), and 17α-ethynylestr-4-en-3β,17β-diacetoxy-10β-ol (**4**) and a known metabolite, 17α-ethynyl-17β-acetoxyestr-4-en-3-one (**5**). The biotransformation of **1** with *Ocimum basilicum* included hydrolysis of the ester group, oxidation of alcohol into ketone, and rearrangement of the hydroxyl group. Thus four major known metabolites were characterized as 17α-ethynyl-17β-acetoxyestr-4-en-3-one (**5**), 17α-ethynyl-17β-hydroxyestr-4-en-3-one (**6**), 17α-ethynyl-3 β-hydroxy-17β-acetoxyestr-4-ene (**7**) and 17α-ethynyl-5α,17β-dihydroxyestr-3-ene (**8**). Biotransformation of **1** with *Azadirachta indica* culture yielded compounds **5** and **6**. Spectroscopic data of compound **8** is being reported for the first time. Structure of compound **6** was unambiguously deduced through single-crystal x-ray diffraction studies.

**Conclusion:**

Biotransformation of an oral contraceptive, ethynodiol diacetate (**1**), by using microbial and plant cell cultures provides an efficient route to the synthesis of a library of new steroids with potential contraceptive properties. These methods can be employed in the production of such compounds with high stereoselectivity.

## Background

Development of efficient, environmental friendly and cost effective routes to synthesize fine chemicals is the need of the day. Biotransformation has been an exciting area of research for decades. Enzymes from various sources, i.e., microorganisms, animal and plant cells, have been employed for carrying out reactions at chemically inaccessible positions of organic compounds. Microbial transformation of steroids has been extensively investigated [1] and various hydroxylated derivatives have been produced on large scale [2]. Cell suspension cultures of plants have also been efficiently employed for the biotransformation of organic compounds, e.g., steroids [3], terpenes [4,5], alkaloids [6] and flavonoids [7]. The reactions carried out by microbial and plant cultures include hydroxylation, oxidation and reduction of alcohols, ketones and C = C bond [8].

*Ocimum basilicum* L. (Lamiaceae) (sweet basil) is found mostly in Asia. It is used as a flavorant in food, perfumery, cosmetics and medicines [9]. There are some reports of biotransformation of chemical compounds with *O. basilicum* culture [10]. *Azadirachta indica* A. Juss. (Meliaceae) (Neem) is native to tropical and semi-tropical Asia. Cell suspension culture of *Azadirachta indica* has also been previously recruited for the structural transformation of dydrogesterone [11].

Ethynodiol diacetate (**1**) is a semi synthetic steroidal drug, used as an oral contraceptive. It inhibits the ovulation process, and serves as a potent progestin. It provides adequate control of menstrual cyclicity in combination with an estrogen, and thus has a complete contraceptive property, even in low doses [12]. The biotransformation of **1** has been previously studied *in vivo* in rhesus monkey [13] and baboon [14], and *in vitro* by rat and human liver cells [15]. Biotransformation of **1** with microbial and plant cell cultures has not been reported earlier. During the current study, we investigated the metabolism of compound **1** with a fungal and two plant cell cultures, which resulted in a number of new **24** and known **58** metabolites.

## Results

### Microbial transformation of ethynodiol diacetate with *C. elegans*

The ^1^H- and ^13^C-NMR chemical shifts of compounds **2**–**5** are presented in Tables 1 and 2, respectively. Other data is presented below:

17α-Ethynylestr-4-en-3β,17β-diacetoxy-6α-ol **(2)**. Colorless amorphous solid (5 mg, 0.5%). ^**1**^**H-NMR** (CD_3_OD, 300 MHz): Table 1, ^**13**^**C-NMR** (CD_3_OD, 75 MHz): Table 2. **EI-MS***m/z* (rel. int., %): 400 (15, *M*^+^), 340 (95), 298 (37), 280 (35), 231 (50), 119 (39), 110 (72), 91 (100), 79 (75), 55 (65). **HREI-MS***m/z* (mol. formula, calcd value): 400.2065 (C_24_H_32_O_5_, 400.2038).

17α-Ethynylestr-4-en-3β,17β-diacetoxy-6β-ol **(3)**. Colorless amorphous solid (10 mg, 1.0%). ^**1**^**H-NMR** (CD_3_OD, 300 MHz): Table 1, ^**13**^**C-NMR** (CD_3_OD, 75 MHz): Table 2. **EI-MS***m/z* (rel. int., %): 400 (15, *M*^+^), 340 (95), 298 (37), 280 (35), 231 (50), 119 (39), 110 (72), 91 (100), 79 (75), 55 (65). **HREI-MS***m/z* (mol. formula, calcd value): 400.2065 (C_24_H_32_O_5_, 400.2038).

**17α-Ethynylestr-4-en-3β,17β-diacetoxy-10β-ol (4)**. Colorless amorphous solid (5 mg, 0.5%). ^**1**^**H-NMR** (CD_3_OD, 300 MHz): Table 1, ^**13**^**C-NMR** (CD_3_OD, 75 MHz): Table 2. **EI-MS***m/z* (rel. int., %): 400 (15, *M*^+^), 340 (95), 298 (37), 280 (35), 231 (50), 119 (39), 110 (72), 91 (100), 79 (75), 55 (65). **HREI-MS***m/z* (mol. formula, calcd value): 400.2065 (C_24_H_32_O_5_, 400.2038).

**17α-Ethynyl-17β-acetoxyestr-4-en-3-one(5)**. Colorless crystalline solid (14 mg, 1.4%). **M. P.** 161–163°C (lit. 161–162°C [16]). ^**1**^**H-NMR** (CDCl_3_, 300 MHz): Table 1, ^**13**^**C-NMR** (CDCl_3_, 75 MHz): Table 2*.***EI-MS**: *m/z* (rel. int., %) 340 (90, *M*^+^, C_22_H_28_O_3_), 298 (37), 231 (47), 119 (39), 110 (69), 91 (100), 79 (75), 55 (65).

**Table 1:** ^**1**^**H-NMR data of compounds 1-8 at 300 (compounds 2,3,4,5,7), 400 (compound 8) and 500 (compound 6) MHz; δ in ppm, *J *and *W ***_***1/2 ***_**in Hz**

**COMPOUNDS**								
**Carbon**	**1**	**2**	**3**	**4**	**5**	**6**	**7**	**8**
1	1.41, 2.05	1.12, 2.07,	1.36, 1.70	1.38, 1.94	1.52, 2.24	1.54, 2.25	1.74, 1.96	1.75, 1.92
2	2.01, 2.27 dt, *J*=13.4, 2.8	1.33, 1.94	1.46, 2.01	1.55, 1.83	2.21, 2.38	2.28, 2.37	1.95, 2.23	1.91, 1.98
3	5.20 br s, *W*_1/2_=19.6	4.05, m (*W*_1/2_=17.4 Hz)	5.20, m (*W*_1/2_=22.8 Hz)	4.01, m (*W*_1/2_=15.6 Hz)	-	-	4.14 m, *W*_1/2_=16.8	5.85d *J* =9.6
4	5.32	5.55, br s (*W*_1/2_=9.7 Hz)	5.55, br s, (*W*_1/2_=17.2 Hz)	5.40, br s (*W*_1/2_=9.37 Hz)	5.81 s	5.81 s	5.37	5.50 d *J*=9.6
5	-	-	-	-	-	-	-	-
6	0.95, 1.68	4.12, br s (*W*_1/2_=9.6 Hz)	4.16, br s, (*W*_1/2_=17.1 Hz)	2.05, 2.41	2.27, 2.49	2.29, 2.45 dt, *J*=14.5, 3.28	1.67, 1.82	1.77, 1.82
7	1.17, 1.80	2.05, 2.65	1.13, 1.87	0.90, 1.75	1.12, 1.83	1.06, 1.82	0.94, 1.73	1.15, 1.78
8	1.25	1.94	1.82	1.72	1.36	1.35	1.25	0.82
9	0.71	0.60	0.65	0.80	0.85	0.86	0.67	1.08
10	1.77	1.80	2.20	-	2.06	2.07 td, *J*=10.5, 4.7	1.75	1.47
11		1.25, 1.84	1.23, 1.85	1.59, 1.67	1.22, 1.91	1.23, 1.88	1.13, 2.02	0.78, 1.51
12	1.67, 1.82	1.65, 1.83	1.66, 1.84	1.66, 1.82	1.70, 1.87	1.63, 1.75	1.27, 2.04	1.54, 1.68
13	-	-	-	-	-	-	-	-
14	1.51	1.50	1.52	1.48	1.54	1.51	1.50	1.42
15	1.31, 1.72	1.37, 1.67	1.27, 1.29	1.37, 1.72	1.33, 1.75	1.27, 1.54	1.32, 1.77	1.28, 1.67
16	1.98, 2.72	2.15, 2.65	2.04, 2.65	2.05, 2.65	1.97, 2.73 ddd, *J*=15, 9.6, 5.7	1.98, 2.27	1.99, 2.70 ddd *J*=15, 6.0, 3.6	1.94, 2.27
17	-	-	-	-	-		-	-
18	0.87, s	0.94, s	0.95, s	0.93, s	0.91, s	0.89, s	0.87, s	0.84, s
20	-	-	-	-	-	-	-	-
21	2.55, s	2.94, s	2.97, s	2.95, s	2.57, s	2.55, s	2.55, s	2.55, s
22	-	-	-	-	-		-	
23	2.02	2.01, s	2.00, s	1.99, s	2.02 s		2.01 s	
24								
25	2.02	2.01, s	2.00, s	1.99, s				

Note: Assignments based on COSY, HMBC and HMQC spectra. Assignments shown without multiplicity means multiplet.

**Table 2:** ^**13**^**C-NMR data of compounds 1–8, MHz; δ in ppm**

**Compounds**								
**C**	**1**	**2**	**3**	**4**	**5**	**6**	**7**	**8**
**1**	27.7	27.0	24.1	34.7	26.6	26.6	25.6	19.2
**2**	34.9	32.5	28.2	29.1	36.5	36.5	34.9	20.8
**3**	70.3	68.0	71.2	68.1	199.8	199.9	67.4	132.0
**4**	119.9	129.0	124.0	128.1	124.7	124.6	124.3	132.4
**5**	144.8	143.0	145.0	143.0	166.3	166.5	142.8	69.7
**6**	31.3	73.5	73.0	32.2	35.4	35.5	32.9	39.7
**7**	25.7	38.3	39.0	33.0	30.7	30.6	31.4	26.6
**8**	41.2	32.2	35.5	37.0	40.7	41.0	41.2	41.2
**9**	49.4	51.4	50.5	55.0	48.9	49.1	49.7	40.9
**10**	41.6	48.9	38.3	70.8	42.5	42.5	41.8	49.4
**11**	25.2	26.5	26.2	20.8	26.2	26.2	25.7	27.8
**12**	32.9	34.2	34.1	34.0	32.8	32.4	32.1	32.7
**13**	47.7	49.0	48.8	48.8	47.5	46.9	47.6	47.0
**14**	47.6	48.9	49.0	49.5	47.6	49.2	47.7	45.9
**15**	23.4	24.2	30.6	24.2	23.4	22.9	23.4	22.9
**16**	37.3	38.2	38.1	38.4	37.2	38.8	37.3	38.9
**17**	84.5	85.9	86.0	86.0	84.3	79.7	84.5	79.9
**18**	13.4	14.0	13.8	14.0	13.4	12.7	13.4	12.7
**20**	83.3	83.0	84.0	84.0	83.2	87.2	83.4	87.6
**21**	74.8	76.5	76.5	76.8	75.0	74.2	74.5	73.9
**22**	169.6	171.5	171.0	171.2	169.5		169.6	
**23**	21.4	21.2	21.2	21.5	21.4		21.5	
**24**	170.9	171.5	172.0	171.2				
**25**	21.4	21.2	21.2	21.5				

### Results of biotransformation of ethynodiol diacetate with *O. basilicum*

The ^1^H- and ^13^C-NMR chemical shifts of compounds **6**–**8** are presented in Tables 1 and 2, respectively. Other data is presented below:

**17α-Ethynyl-17β-hydroxyestr-4-en-3-one(6)**. Colorless crystalline solid (20 mg, 3.3%). **M. P.** 201–203°C (lit. 203–204°C [17]). ^**1**^**H-NMR** (CDCl_3_, 500 MHz): Table 1, ^**13**^**C-NMR** (CDCl_3_, 150 MHz): Table 2*.***EI-MS**: *m/z* (rel. int., %) 298 (81, *M*^+^, C_20_H_26_O_2_), 231 (71), 160 (40), 135 (44), 110 (85), 91 (100), 79 (77), 55 (60). **Crystal data**: C_20_H_26_O_2_, Mr = 298.41, Orthorhombic, space group P2_1_2_1_2_1_, *a* = 6.5463(5) Å , *b* = 12.1646(10) Å , *c* = 20.7743(17)Å*, α*, *β*, *γ* = 90^o^, *V* = 1654.3(2) Å^3^ , *Z* = 4, *ρ*_*calc*_ = 1.198 mg/m^3^, *F*(000) = 648, *μ* (Mo Kα) = 0.71073 Å, max/min transmission 0.9881/ 0.9669, crystal size 0.45 x 0.17 x 0.16, 1.94° < θ< 25.5°, 9821 reflections were collected, of which 3,601 reflections were judged observed (*R*_int_ = 0.0274). The *R* values were: R1 = 0.0367, wR2 = 0.0860 for I > 2σ(I), and R1 = 0. 0.0424, wR2 = 0.890 for all data; max/min residual electron density: -0.148 eA/-0.148 eA^−3^. The structure was solved by the direct methods, expanded by using Fourier transformation techniques [18] and refined by a full-matrix least-square calculation on *F*^*2*^ with the aid of SHELXL97 program [19]. Crystallographic data for compound **6** has been deposited in the Cambridge Crystallographic Data Center. The crystallographic information can directly be obtained free of charge from CCDC data center (CCDC 837461 reference code).

17α-Ethynyl-3β-hydroxy-17β-acetoxyestr-4-ene **(7)**. Colorless amorphous solid (3.5 mg, 0.58%). ^**1**^**H-NMR** (CDCl_3_, 300 MHz): Table 1, ^**13**^**C-NMR** (CDCl_3_, 75 MHz): Table 2*.***EI-MS**: *m/z* (rel. int., %) 342 (100, *M*^+^, C_22_H_30_O_3_), 255 (5), 185 (4), 145 (15), 105 (30), 91 (43), 81 (51), 55 (49).

17α-Ethynyl-5α,17β-dihydroxyestr-3-ene **(8)**. Colorless amorphous solid (2.7 mg, 0.45%). ^**1**^**H-NMR** (CDCl_3_, 400 MHz): Table 1, ^**13**^**C-NMR** (CDCl_3_, 100 MHz): Table 2. **EI-MS**: *m/z* (rel. int., %) 300 (22, *M*^+^, C_20_H_28_O_2_), 282 (37), 199 (63), 149 (73), 91 (100), 81 (89), 55 (86).

### Results of biotransformation of ethynodiol diacetate with *A. indica*

Biotransformation of **1** with *A. indica* afforded two known metabolites **5** and **6**, which have been discussed earlier.

## Discussion

In the current study, biotransformation of ethynodiol diacetate (**1**) C_24_H_32_O_4,_ with *C. elegans* is being carried out for the first time, affording three new **2**–**4** and a known **5** metabolite. Biotransformation of **1** was also investigated with cell cultures of *O. basilicum* yielding four known metabolites **5**–**8**. Substrate **1** was also subjected to biotransformation with *A. indica* and two known metabolites **5** and **6** were obtained.

The molecular formula for metabolite **2** (C_24_H_32_O_5_) was obtained from the HREI-MS [*M*^*+*^*m/z* 400.2065 (calcd 400.2038)], which was 16 a.m.u. higher than the substrate **1**. The compound was found to be UV inactive. The IR spectrum showed the presence of an ester carbonyl (1742 cm^-1^), and an -OH (3433 cm^-1^) functional groups.

The 16 a.m.u. increment in the *M*^+^ of the metabolite **2**, as compared to substrate **1**, could be attributed to the addition of an oxygen atom. The ^1^H-NMR of **2** (Table 1) showed a methyl singlet at δ 0.94, and a six-proton singlet (2 x CH_3_) at δ 2.01. This suggested that both ester groups remain intact. Therefore the change was assumed to be the hydroxylation of substrate **1**. A downfield proton signal at δ 4.12 (br. s., *W*_1/2_ = 9.6 Hz) with its corresponding carbon at δ 73.5 appeared in the HSQC spectrum. Another downfield proton signal at δ 4.05 (m, *W*_1/2_ = 17.4 Hz) showed HMBC correlation with the ester carbonyl carbon (δ 171.5). This proton was therefore assigned to H-3. The H-3 showed COSY interaction with the olefinic proton (δ 5.55, br. s., *W*_1/2_ = 9.7 Hz), which was assigned to H-4. H-4 in turn, showed a weak allylic coupling with the hydroxyl-bearing methine proton (δ 4.12) in COSY spectrum. This suggested that the hydroxylation had occurred at C-6 of the steroidal skeleton. This was further confirmed by the HMBC correlations of H-6 (δ 4.12) with C-4 (δ 129.0), and C-10 (δ 48.9). H-8 (δ 1.94) showed NOESY interactions with H-6 (δ 4.12) indicating that the C-6 proton was β-oriented, thus the *geminal* hydroxyl group was α-oriented. The structure of **2** was thus deduced as 17α-ethynylestr-4-en-3β,17β-diacetoxy-6α-ol.

Metabolite **3** had the same molecular composition (C_24_H_32_O_5_) as that of **2**, as deduced from the HREI-MS [*M*^*+*^*m/z* 400.2065 (calcd 400.2038)]. The compound was found to be UV inactive. The IR spectrum showed absorptions for the ester carbonyl (1740 cm^-1^), and hydroxyl (3433 cm^-1^) groups.

**Figure 1:**
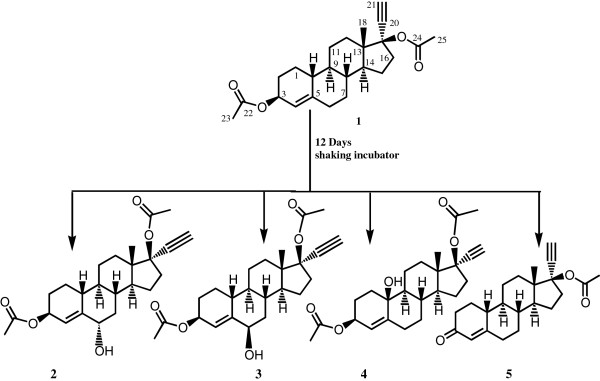
**Biotransformation of ethynodiol diacetate(1) with *Cunninghamella elegans.***

The ^1^H- and ^13^C-NMR spectra of metabolite **3** were very similar to **2**. A downfield hydroxyl-bearing methine proton signal at δ 4.16 (br. s., *W*_1/2_ = 17.1 Hz) with its corresponding carbon at δ 73.0 appeared in the spectra of **3**. The C-4 olefinic proton (δ 5.55, br. s., *W*_1/2_ = 17.2 Hz) showed a weak allylic coupling with the hydroxyl-bearing methine proton (δ 4.16). This suggested that the hydroxylation had occurred at C-6 of the steroidal skeleton. The NOESY spectrum did not show any correlation between H-6 (δ 4.16), and H-8 (δ 1.82, *axial*). Therefore it was assigned an *equatorial* orientation (α-orientation). The rest of the proton and carbon values were distinctly similar to metabolite **2**. Metabolite **3** was characterized as a new compound (17α-ethynylestr-4-en-3β,17β-diacetoxy-6β-ol) Figure 1.

The molecular composition C_24_H_32_O_5_ for metabolite **4** was obtained from the HREI-MS [*M*^*+*^*m/z* 400.2065 (calcd 400.2038)], 16 mass units higher than substrate **1**. The compound was found to be UV inactive, suggesting lack of any conjugated system. The IR spectrum showed the presence of ester carbonyl (1740 cm^-1^), and -OH (3433 cm^-1^) groups.

The molecular formula and the IR spectrum of **4** suggested the hydroxylation of substrate **1**, but the ^1^H-NMR spectrum (Table 1) of **4** did not show any downfield hydroxyl-bearing methylene proton signal. A downfield multiplet at δ 4.01 (*W*_1/2_ = 15.6 Hz), and a broad singlet at δ 5.40 (*W*_1/2_ = 9.4 Hz) were vicinally coupled in the COSY 45^o^ spectrum. These were assigned to H-3 (δ 4.01), and H-4 (δ 5.40), with corresponding carbons at δ 68.1 and 128.1, respectively. A downfield quaternary carbon signal at δ 70.8 was HMBC correlated with H-4 (δ 5.40). The only position thus available for hydroxylation was C-10. The hydroxyl group at C-10 was assigned axial orientation in correspondence with those of previously reported compounds, 3-ethyl-6*β*,17*β*-dihydroxy-18,19-dinor-17*α* -pregn-4-en-20-yn-3-on [20], 13-ethyl-6*β*,10*β*,17*β*-trihydroxy-18,19-dinor-17*α* -pregn-4-en-20-yn-3-on [21], and 10*β*-hydroxy-19-nor-testosterone [22]. The rest of the spectrum closely resembled with the substrate **1**, as well as metabolites **2** and **3**. The ^13^C-NMR spectrum of **4** had one CH less than the substrate, and an additional downfield quaternary carbon (δ 70.8) which further supported the proposed structure, 17α-ethynylestr-4-en-3β,17β-diacetoxy-10β-ol for metabolite **4**.

The *M*^+^ of compound **5** (*m/z* 340, C_22_H_28_O_3_), 43 amu less than compound **1**, suggested the loss of an acetyl moiety, either from C-3 or C-17. The compound showed florescence under the UV light indicative of the conversion of the ester into an unsaturated ketone, through hydrolysis followed by oxidation. This also confirmed that the ester at C-3 had been hydrolyzed, while C-17 ester remains intact. This was confirmed with the help of ^1^H- and ^13^C-NMR spectra. The broad singlet at δ 5.20 (H-3) was absent in the ^1^H-NMR spectrum of **5**. ^13^C-NMR showed the presence of a new ketonic carbonyl signal at δ 199.8 (C-3) and the absence of the ester carbonyl at δ 170.9 (C-24). The compound was thus characterized as norethisterone acetate. It is a potent oral progestational agent. Compound **5** has been reported earlier as an *in vitro* metabolite of ethynodiol diacetate by rat and human liver cells [15].

The EI-MS of **6** (C_20_H_26_O_2_) showed the *M*^+^ at *m/z* 298. The 85 amu decrease in molecular weight suggested the hydrolysis of both the ester groups. The UV florescence indicated the oxidation of the hydroxyl group, formed through hydrolysis of C-3 ester followed by oxidation into the corresponding α, β-unsaturated ketone.

**Figure 2:**
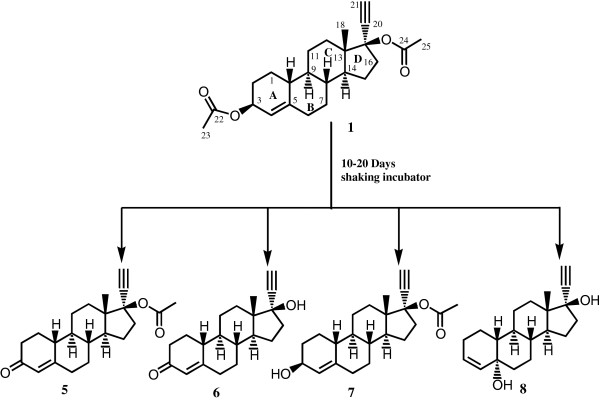
**Biotransformation of ethynodiol diacetate(1) with cell suspension cultures of *Ocimum basilicum *(compounds 5–8, in 20 days) and *Azadirachta indica *(compounds 5 and 6, in 10 days).**

**Figure 3:**
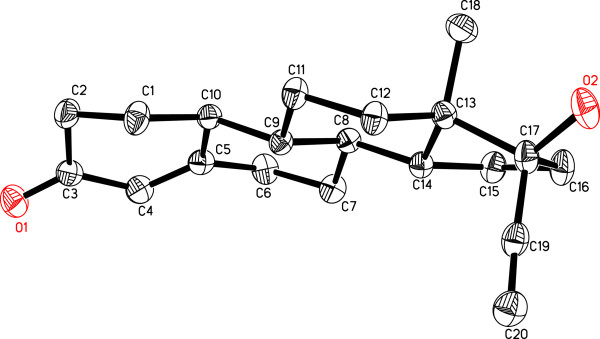
**Computer-generated ORTEP diagram of metabolite 6.** Hydrogens are omitted for clarity.

The *M*^+^ of **6** was 42 amu less than **5**, suggesting the hydrolysis of C-17 ester group. The rest of the spectrum was in close correspondence with metabolite **5**. The compound was characterized as norethisterone. It is a progestin used as oral contraceptive pills. Single-crystal X-ray diffraction analysis was carried out to establish the structure of compound **6** (Figures 2 and 3). The ORTEP diagram of **6** (Figure 3) showed four *trans* fused rings A, B, C, and D with *chair*, *half chair*, *chair*, and *envelop* conformations, respectively. The C-17 -OH and acetylene groups existed in *pseudo-equatorial* and *pseudo-axial* orientations, respectively. All the bond angles and lengths were within the normal range. The figure was plotted with the aid of ORTEPII program [23]. Earlier *in vitro* incubation of **1** with rat and human liver cells by Freudenthal *et. al*. has led to the formation of **6**[15].

The EI-MS of **7** (C_22_H_28_O_2_) showed the *M*^+^ at *m/z* 342, 2 amu higher than **5**, attributed to the hydrolysis of the C-3 ester into an -OH which did not oxidized into a ketone, as in **5**. The ^1^H-NMR also showed a broad singlet at δ 4.14 (*W*_1/2_ = 16.8 Hz, H-3), while other broad singlet at δ 5.20 (proton geminal to ester group in **1**) was absent. The spectrum also showed a singlet for methyl group at δ 2.01 (H-23), further indicating that the ester at C-17 remained intact. The rest of the spectrum was distinctly similar to substrate **1**. Compound **7** was unambiguously identified as 17α-ethynyl-3β-hydroxy-17β-acetoxyestr-4-ene. Metabolite **7** has earlier been obtained from the *in vitro* biotransformation of ethynodiol diacetate (**1**) with rat and human liver cells [15].

The *M*^+^ of compound **8** (C_20_H_28_O_2_) appeared at *m/z* 300 (EI-MS). The ^1^H-NMR spectrum of **8** showed two olefinic proton doublets at δ 5.85 (*J*_3,4_ = 9.6 Hz) and 5.84 (*J*_4,3_ = 9.6 Hz). The olefinic protons belonged to adjacent carbon atoms as inferred from the COSY spectrum and assigned to H-3 and H-4, respectively. The spectrum was also devoid of any hydroxyl-bearing methine proton signal. The ^13^C-NMR spectrum of **8** showed no ketonic carbonyl signal, but two tertiary hydroxyl carbon signals, appeared at δ 69.7 and 79.9. The signal at δ 79.9 was assigned to C-17, in comparison with metabolite **6**. Carbon resonating at δ 69.7 was HMBC correlated with the proton at δ 5.85 (H-4) and thus assigned to C-5. The metabolite **8** was thus identified as 17α-ethynyl-5α,17β-dihydroxyestr-3-ene. Compound **8** was earlier obtained from the photosensitized oxidation of 19-nor-17α-pregn-4-en-20-yn-17-ol [24]. The spectroscopic data of this compound was not reported previously.

### Experimental

#### General

Ethynodiol diacetate (**1**) was purchased from Sigma-Aldrich. Thin layer chromatography was carried out on precoated plates (Silica gel, Merck, PF_254_). Column chromatography (CC) was performed by using silica gel (E. Merck, Germany). ^1^H- and ^13^C-NMR spectra were recorded in CDCl_3_ and CD_3_OD on Bruker Avance-NMR spectrometers. The chemical shifts (δ values) are presented in ppm and the coupling constants (*J* values) are in Hertz. JEOL (Japan) JMS-600H mass spectrometer was used for recording EI-MS in *m/z* (rel. %). Single-crystal X-ray diffraction data was collected on Bruker Smart APEX II, CCD 4-K area detector diffractometer [25]. Data reduction was performed by using SAINT program. The structure was solved by direct methods [26], and refined by full-matrix least squares on F2 by using the SHELXTL-PC package [27]. The figures were plotted with the aid of ORTEP program [20].

#### Microbial and callus cultures

Culture of *Cunninghamella elegans* was purchased from NRRL (1392), grown on Saboraud dextrose agar (SDA). The culture medium for *C. elegans* was prepared by dissolving glucose (40 g), yeast extract (20 g), peptone (20 g), NaCl (20 g), KH_2_PO_4_ (20 g) and glycerol (40 mL) in distilled water (4.0 L).

Plant material of *Ocimum basilicum* and *Ocimum sanctum* were obtained from the greenhouse facility of the H. E. J. Research Institute of Chemistry, University of Karachi. Callus culture of the plant was derived from young leaves which were cultivated in 300 mL jars, containing 25 mL of Murashige and Skoog (MS) media [28], each supplemented with 2% sucrose, 0.5 mg/L 2,4-diphenoxy acetic acid (2,4-D), 2.5 mg/L naphthalene acetic acid anhydride (NAA), 0.01 g/L ascorbic acid and solidified by 0.6% agar at 25 ± 1°C in the dark.

The callus culture of the *Azadirachta indica*, also obtained from the greenhouse facility of the H. E. J. Research Institute of Chemistry, was established from young leaves, cultivated in 300 mL jars having 25 mL of Murashige and Skoog media [28], enriched with sucrose (30 g/L), 3-indole butyric acid (4 mg/L), 6-benzyl aminopurine (1 mg/L), and agar (6 g/L) at 25 ± 1°C under complete darkness.

#### Fermentation of ethynodiol diacetate (1) with C. elegans and purification of metabolites

4.0 L of culture medium for *C. elegans* was prepared as described earlier and distributed evenly among 40 Erlenmyer flasks (100 mL each). The flasks were plugged with cotton swab and sterilized in an autoclave at 121°C for 15 minutes. Spores of the fungus were transferred into 10 flasks under sterilized conditions in a laminar flow cabinet to prepare the seed flasks. These innoculated flasks were kept on a rotary shaker for two days and then the seed flasks were used to inoculate the remaining 30 flasks with spores of *C. elegans* which were again kept on shaker for incubation. After enough growth, the substrate (**1**, 1.0 g) dissolved in acetone (20 mL), was transferred equally to all the flasks under sterilized conditions. The flasks were again kept on shaker for fermentation and time course study was conducted by harvesting the content of one flask and checking the extent of transformation on TLC. The fermentation was continued for 12 days. The culture medium was then filtered to separate mycelium from broth, and filtrate was extracted with dichloromethane (DCM) (4 L × 3). The organic phase was collected, dried (Na_2_SO_4_), and concentrated *in vacuo* to obtain a brown gum (1.6 g). This gum was fractionated on silica gel with petroleum ether and ethyl acetate as mobile phase. Main fractions were subjected to silica gel column chromatography by using gradient eluent systems of pet. ether/ ethyl acetate to obtain metabolites **2**, **3** and **4** at 30% and metabolite **5** at 40% ethyl acetate in pet. ether.

#### Fermentation of ethynodiol diacetate (1) with O. basilicum and purification of metabolites

Cell suspension cultures were derived from static cultured calli in Erlenmeyer flasks (1 L), containing 400 mL of the culture medium. The flasks were placed on a shaker (100 rpm) with a 16 hours photoperiod at 25 ± 1°C for 15 days of pre-culturing. A solution of compound **1** (600 mg) in acetone (100 mg/mL) was added to each flask through a 0.2 *μ*M membrane filter (millipore) and the flasks were again placed on shaker for 20 days. Negative (containing only plant cell suspension culture) and positive (compound **1** in the medium) controls were also prepared. Time course study was carried out on a daily basis and the extent of bioconversion was analyzed by TLC. The fermentation media was filtered and filtrate was extracted thrice with DCM, dried over anhydrous Na_2_SO_4_, and evaporated *in vacuo*. The extract (2.0 g) was subjected to fractionation with 10% gradient of pet. ether/acetone (P.E./Ac.), followed by further column chromatography to obtain metabolites **5** (8:2 P.E./Ac.), **6**, **7** (7:3 P.E./Ac.) and **8** (6:4 P.E./Ac.) in appreciable quantities.

#### Fermentation of ethynodiol diacetate (1) with A. indica and purification of metabolites

Cell suspension culture was derived from static calli, cultured in Erlenmeyer flasks (1 L), each containing 400 mL of the Murashige and Skoog media, supplemented with ingredients as mentioned above, except BA and agar. After 20 days of pre-culturing on a shaker (100 rpm) and 16 hours of photoperiod at 25 ± 1°C, a solution of substrate (100 mg in 1 mL of acetone) was added to each flask through a 0*.*2 *μ*M membrane filter and the flasks were placed on a shaker for 10 days. The time course study was performed and the course of biotransformation was monitored by TLC. Positive and negative controls were also run along with the main experiment in order to differentiate the transformed products from metabolites. After 10 days of incubation, the cells and the media were separated by filtration. The filtrate (~2 L) was extracted with CH_2_Cl_2_ (3 × 2 L) at r. t. The combined extract were dried over anhydrous Na_2_SO_4_, and concentrated *in vacuo*, which afforded a brown residue (1.1 g). The transformed metabolites were isolated from this gummy crude by using repeated column chromatography (silica gel) with petroleum ether/EtOAc gradient, affording compounds **5** (6:4 P.E./EtOAc) and **6** (1:1 P.E./EtOAc).

## Conclusion

In conclusion, the biotransformation of oral contraceptive ethynodiol diacetate (**1**) with *C. elegans*, *O. basilicum* and *A. indica* was investigated for the first time which provided an efficient route to several metabolites. Biotransformation of **1** with *C. elegans* led to the formation of three new and one known metabolites, while biotransformation with cell suspension cultures of *O. basilicum* and *A. indica* afforded four known metabolites. Metabolite **5** was obtained in all three experiments. Single-crystal X-ray structure of metabolite **6** and spectroscopic data of metabolite **8** are being reported here for the first time. Metabolites **5**, **6** and **7** were reported previously as *in vitro* metabolites of ethynodiol diacetate (**1**) from rat and human liver cells.

## Competing interests

Two of the authors, S. Zafar and H. A. Kayani, acknowledge the Higher Education Commission, Pakistan, for providing financial support through the HEC indigenous Ph. D. scholarship program.

## Authors’ contributions

SZ Carried out the microbial transformation by using *Cunninghamella elegans*, purified all the metabolites and solved the spectroscopic data. SY conducted the single-crystal X-ray crystallographic studies. HAK carried out the biotransformation by using cell cultures of *Ocimum basilicum*. Saifullah carried out the biotransformation by using cell suspension cultures of *Azadirachta indica*. SK helped in the biotransformation experiments with plant cell cultures. AAM helped in the preparation of the manuscript. MIC conceived the original study, supervised the research, helped in solving the spectroscopic data, and finalized the manuscript. All authors read and approved the final manuscript.
